# Perils and Promises of Pathogenic Protozoan Extracellular Vesicles

**DOI:** 10.3389/fcimb.2020.00371

**Published:** 2020-08-14

**Authors:** Joshua Seun Olajide, Jianping Cai

**Affiliations:** ^1^State Key Laboratory of Veterinary Etiological Biology, Key Laboratory of Veterinary Parasitology of Gansu Province, Lanzhou Veterinary Research Institute, CAAS, Lanzhou, China; ^2^Centre for Distance Learning, Obafemi Awolowo University, Ile-Ife, Nigeria

**Keywords:** protozoa, extracellular vesicles, exosomes, effects, host cells, stressor

## Abstract

Extracellular vesicles (EVs) are membranous structures formed during biological processes in living organisms. For protozoan parasites, secretion of EVs can occur directly from the parasite organellar compartments and through parasite-infected or antigen-stimulated host cells in response to *in vitro* and *in vivo* physiological stressors. These secreted EVs characteristically reflect the biochemical features of their parasitic origin and activating stimuli. Here, we review the species-specific morphology and integrity of parasitic protozoan EVs in concurrence with the origin, functions, and internalization process by recipient cells. The activating stimuli for the secretion of EVs in pathogenic protozoa are discoursed alongside their biomolecules and specific immune cell responses to protozoan parasite-derived EVs. We also present some insights on the intricate functions of EVs in the context of protozoan parasitism.

## Introduction

Protozoa are single-celled eukaryotes with enormous structural complexity and diversity. The study of parasitic protozoa began in the 17th century (Cox, 2002) and, at the least, there are about 90 etiologic species of important human parasitic diseases (Coakley et al., 2015), while several other species affect economically important animals (Taylor, 2000). Pathogenic and amphizoic protozoa (Gonçalves and Ferreira, 2019) are dispersed within phyla amoeba, apicomplexa, metamonada, parabasalia and kinetoplastida (Szempruch et al., 2016a), which are known to cause a wide range of important diseases such as amoebiasis, malaria, babesiosis, toxoplamosis, leishmaniasis, trypanosomiasis, cryptosporidiosis, trichomoniasis, giardiasis, neosporosis, theileriosis, etc. Over the years, there have been continuous investigations on protozoan parasites' sub-cellular components, organellar structures, secretory/excretory molecules, and, recently, extracellular vesicles (Yanez-Mo' et al., 2015). As revealed from several studies, a substantial amount of parasitic molecules are carried by EVs secreted directly by parasites (Mantel and Marti, 2014), parasite-infected host cells (Atayde et al., 2015), and host cells stimulated by parasite antigens (Wu et al., 2019).

The trajectories of cellular and molecular involvement of pathogenic protozoan EVs during infection are being unfolded (Li et al., 2018a; Correa et al., 2019). Nevertheless, we have tasked ourselves on EVs that are directly secreted by pathogenic protozoa and those of protozoan parasite origin from parasitized host cells in the case of *Plasmodium* species. Briefly, we discuss the biogenesis of protozoan parasites EVs and the activating physico-chemical stressors that are involved in the formation and release of these vesicles. Intrinsic aspects of vesicular cargo content and functions are also discoursed with pathophysiological effects of pathogenic protozoan EVs (PPEVs) on the host cells and protozoan parasites after fusion and/or internalization.

## PPEVs: Formation, Characterization, and Subcellular Origin

EVs are diverse, distinct membrane-bound structures that are formed and discharged as instruments of structural re-organization, stress response and survival among protozoa (Zhang et al., 2014). The release of EVs occurs either through direct budding from cell membranes (ectocytosis) or through the release of preformed vesicles from cellular compartments (exocytosis) (Sadallah et al., 2010). The systemic secretion of EVs is evolutionarily conserved among living organisms (Yanez-Mo' et al., 2015; Sampaio et al., 2017), and it is a constitutive cellular processes among protozoan parasites (Deolindo et al., 2013; Kehrer et al., 2016). Largely, the secretion of vesicles by parasitic protozoa maintains parasite-defensive mechanisms (Wowk et al., 2017), initiation of parasite infection and stronger interaction with host cells (Da Silveira et al., 1979; Ramirez et al., 2017; Moreira et al., 2019).

On the basis of biogenesis and size, and with respect to protozoan parasites, EVs are broadly classified into exosomes, ectosomes [microparticles or microvesicles (MVs)], and apoptotic bodies (Dong et al., 2019; Cronemberger-Andrade et al., 2020). Ectosomes are vesicles formed from protrusions on the plasma membrane (PM), while inward budding of endosomes forms microvesicle bodies (MVBs), which exocytically fuse with the plasma membrane to form exosomes (Lozano et al., 2017). Apoptotic bodies are formed through the condensation and segregation of the nucleus and the deterioration and blebbing of PM (Torró et al., 2018). Incidentally, simultaneous secretion of exosomes and plasma membrane blebs has been predicted among *Leishamania* spp (Montaner et al., 2014). The biogenesis and classification of exosomes and other EVs have been expertly reviewed in depth by Garcia-Silva et al. (2014), Colombo et al. (2014), and Gavinho et al. (2018). The internal volume of an exosome ranges between 20 and 90 nm^3^ with a capacity to lodge an estimated 100 proteins and 10,000 nucleotides, values that should be higher in ectosomes and apoptotic bodies (Torró et al., 2018). EVs generally are between 20 nm and 1 μm (Mantel and Marti, 2014) but larger vesicles have been found among protozoan parasite species (Barbosa et al., 2018) (Table 1). Unlike the usual lipid bi-membranous layer of EVs, *Leishmania major* promastigote exosomes have their content protected by a phospholipid membrane (Leitherer et al., 2017), and vesicles of *Plasmodium falciparum*-infected red blood cells (*P. falciparum*-iRBCs) are mainly unilamellar (Sisquella et al., 2017).

**Table 1:** Preparation and description of pathogenic protozoan-derived Evs.

**Clade/species (strain)**	**Vesicle type**	**Activatory stimuli**	**Isolation methods**	**Sub-cellular origin**	**Size** **(mean or range)**	**Major vesicular** **content**	**References**
**Amoeba**							
*E. histolytica* trophozoite (HM-1-IMSS)	Cytoplasmic vesicles EDG	Liver lesion/TYI-SS MD	?	Plasma membrane, cytoplasm cell periphery	0.1–1.0 μm 50–200 nm	Cationic and actin proteins	Chavez-Munguia et al., 2004
*A. castellanii* trophozoite (ATTC-30234)	EVs	Glucose MD	2, 5	?	31.9–467 nm 33.7–303.2nm	Serine protease, metalloproteinase, phospholipid, sterylesters, free fatty acids,	Gonçalves et al., 2018
*A. castellanii*	Exosome-like vesicles	PYG MD/page's Neff's saline	4,3,5	?	166.7 nm	IUNH, carboxylic ester hydrolase, peroxidase, aminopeptdase	Lin et al., 2019
**Apicomplexa**							
*P. berghei* (ANKA)	Microparticles	Parasitized RBCs	1,5	iRBCs	150–250 nm	?	Couper et al., 2010
*P. berghei* sporozoite and gametocyte	Secretory vesicles	Ookinete medium	1	Anterior end of parasite		Pantothenate transporter^1^ osmiophilic bodies factor like G377, TRAP	Kehrer et al., 2016
*P. falciparum* (3D7)	EVs		4,2,5,7	infected RBCs	100–400 nm	Ago2, miRNA	Mantel et al., 2016
*P. falciparum*	Exosome-like vesicles	CM 2–4% haematocrit	1,6	Maurer's cleft/infected RBCs	~70 nm	PfPTP2, DNA	Regev-Rudzki et al., 2013
*P. falciparum* (NF54)	EVs	iRBCS	4,2,7	iRBCs	50–350 nm	(ds) gDNA, tRNA, 5sRNA miRNA (hsa-miR-451a)^+^,mRNA, DNA-binding protein H3, H4	Sisquella et al., 2017
*P. falciparum* (3D7 & CS2)	Microvesicles	iRBCs	4,2,7	iRBCs	100–250 nm	PVM, RESA, SBP1, Exp1, parasite invasion proteins	Mantel et al., 2013
*P. yoelii*(17X)	Exosomes	Mice-infection	4	iReticulocytes	~56.8 nm	serine-repeat antigen, MZ surface protein 1&9, protease hsps, enzymes	Martin-Jaular et al., 2011
*P. falciparum* (3D7)	EVs	Modified RPMI	1,4,5	Infected cell	~100	Glycophorine, CD63, PfMSP1, lactate dehydrogenase	Correa et al., 2019
*N. caninum* tachyzoite (Nc-1)	Vesicles	RPMI, 2% Exo-FBS	1,2,5	Parasite surface	50–150 nm	Functional proteins of ribos-omes, metabolism, RNA transport, hsp70&90, proteosome	Li et al., 2018c
*T. gondii* tachyzoite (ME49 & RH)	Exosomes	DMEM without serum	2,8	?	10–150 nm	hsp70,surface antigen 1 (SAG1)	Li et al., 2018a
*T. gondii* tachyzoite (RH)	EVs	RPMI without FBS	1,2,9	Membrane sur-face of parasite	138.2–171.9 nm	15–70 kDa protein spectrum	Silva et al., 2018
*T. gondii* tachyzoite (RH)	EVs	FBS free DMEM	1,2,8	?	130.8 ± 3.7 nm	Celullar, interaction, metabo-lic, regulation, response protei	Wowk et al., 2017
*T. gondii* tachyzoite (RH) highly virulent	Exosomes ectosomes	Sterile PBS at 37°C	4,5	Apical & posterrior end, PM	50–200 nm	MIC, ROP, GRA, phosphatase, metabolic proteins	Ramírez-Flores et al., 2019
***Kinetoplastida***							
*T. cruzi* epimastegote (Y)	Vesicles	Acetate, NaCl CaCl_2_	4,5	FP, PM evagination	0.5 μm	Glycoproteins	Da Silveira et al., 1979
*T. cruzi* blood trypomastigote (Tcl)	EVs	FBS free RPMI	1,2,8		136.33 ± 86.3 nm	TcTASV-C secreted virulence factor	Caeiro et al., 2018
*T. cruzi* trypomasteg-(Tulahuen)	Exosomal vesicles, TESA EVs	FBS free EMEM	1,5,7	PM	60–100 nm	TESA, trans-sialidases, protease gp63, TolT, MASP, mucin-like protein TASV-C	Bautista-lópez et al., 2017
*T. cruzi* Epimastegote (Dm 28c clone)	Vesicles, reservosomes golgi-like vesic	Serum free/1% FBS in RPMI	4,5	Golgi complex, cytostome, FP	20–200 nm	TcPIWI-trypomastegote protein tsRNAs	Garcia-Silva et al., 2014
*T. cruzi*: E,P,A (PAN4 Tcl)	Vesicles	RPMI with 10% free-EV IFCS	5	Parasite surfa-ce, flagellum	50–100 nm	Mucin, MASP with signal peptide (SP)	Lozano et al., 2017
*T. cruzi* (Y, CL-14, YuYu)	Vesicles	RPMI with 5% glucose	1,2,9	membrane sur-face	≤ 200 nm	Proteins and terminal α-galact-osyl residues	Nogueira et al., 2015
*T. cruzi*: E, MT (Dm28c^27^ clone)	Vesicles, MVs, LVs	DMEM without FBS	2,5	PM, FP	100–200 nm	Host-parasite interaction, signaling, transcription, hsps, chaperons, proteolytic proteins	Bayer-Santos et al., 2013
*T. cruzi* : T. (Y, CL-Brener)	Vesicles	HBSS	4,5	Cell body, FP	40–500 nm	Acid and alkaline phosphatases	Nievas et al., 2018
*T. cruzi*: T. (YuYu and Y)	EVs	DMEM with 2% glucose	1,2,10	?	2–3 μm	Transsialidases. MASPs, gp63 tubulin, hsp, mucins, proteases	Ribeiro et al., 2018
*T. cruzi*: E,T (clone Dm 28c)	EVs	FBS freeDMEM/TAU3AAG	2,5	?	?	rRNA, tRNA, CCD, snoRNA and snRNA	Bayer-Santos et al., 2014
*T. brucei gambiense* (Feo, Ok, and Biyamina)	Microvesicles	Secretion medium	1,2,5	PM, FP	50–100 nm	Degradation, nucleotide metabolism, folding protein	Geiger et al., 2010
*T. b. gambienese*(KETRI2482)	Nanotubules/EVs	RNAi-α-KDE1 complement active FBS, inaactvated serum	2,5	FP	70–165 nm	vSG, hsp70, glycerol kinase, matrix glycosomes, mitochondrial membrane protein	Szempruch et al., 2016b
*T. brucei*: procyclic	Exosomes	Trans-splicing inhibition (Vp36 silencing)	2,5,7	FP, membrane nanotubules	50–200 nm	SL RNA-associated proteins, p22, p27, and p58	Eliaz et al., 2017
*L. infatum*: P. (clone)	Vesicles	Miltefosine/apoptosis indicers, G418	5,3	?	30–100 nm	gp63, ribosomal protein, hsp70 elongation factor-1α, beta tubulin, β-fructofuranosidases^1^	Santarém et al., 2013
*L. donovani, L. major, L. mexicana*	Microparticles, (Exosomes, vesicles)	Neutral and acidic medium	4	PM, FP, phagol ysosome	30–70 nm	TESA, trans-sialidases, protease transport, metabolic protein	Silverman et al., 2010a
*L. donovani* HSP100–^/^- and wildtype)	Exosomes	RPMI with HEPES, MES	4,2,7	?	?	hsp100, 90, 70.4, gp63, histone, chaperonin proteins	Silverman et al., 2010b
*L. major*	Exosome-like	Insect	11	Membrane sur	50–120 nm	GP63, calpain-like cysteine peptida	Atayde et al., 2015
*L. infantum P*	Vesicles	Gut		Face,FP, MVB		se, HSP70, tryparedoxin peroxidase surface antigen protein	
*L. infantum* P,A	Exosome	RPMI pepton	1,4	?	122 ± 56 nm	HSP70, HSP83/90,	Castelli et al., 2019
	Vesicles	Yeast			115 ± 65 nm	Acetylcholinesterase	
*L. amazonensis* P (-M2269)	Evs	RPMI/20% glucose	2,4	Whole body	180 nm	gp63, LPG	Barbosa et al., 2018
*T. vaginalis* (B7RC2&jtwild)	Microvesicle-like structure	Serum free TYM with CaCl_2_	1,2,3	PM, Flagellum	100–1,000 nm (>1 μm)	Metabolic enzymes, ribosomal, cytoskeletal, endoplasmin Memebrane vacoule proteins	Nievas et al., 2018
**Parabasalia**							
*T. vaginalis*
(B7RC2, G3, T1, RU38)	Exosome	TYM without serum	2,5,7	Large vesicular bodies	50–100 nm	Small RNAs, tetraspanins, Alix, Rabs, hsp70, signaling and metabolic proteins	Twu et al., 2013
**Diplomonadida**							
*G. intestinalis*	Microvesicles	Serum free YiS with CaCl_2_	4,5	Trophozoite	201.4 nm	Nuclear, surface, cytoskeletal proteins, and chaperones	Evans-Osses et al., 2017

*1. Centrifugation 2. Filtration, 3. Concentration by ultrafiltration/high molecular weight cut-off filter 4. Sequential/serial centrifugation 5. Ultracentrifugation, 6. Buoyant density on Optirep gradient fractionation 7. Buoyant density on sucrose gradient fractionation 8. precipitation by exo-prep kit 9. Gel exclusion chromatography, 10. Size exclusion chromatography 11. Dissection/Suspension in PBS FP, flagellar pocket, PM, plasma membrane T, trypomastegote, E, emastegote A, amastegote*,

1 *putative, CM, culture medium; MD, medium; EDG, electron dense granules; IUNH, inosine-uridine- preferring nucleoside hydrolase family protein; SAG, surface antigen protein; MIC, microneme proteins; RESA, trypomastigote excreted-secreted antigens; SBP1, skeleton binding protein 1; PVM, Parasitophorous vacuole membrane protein; GRA, dense granule antigens; ROP, Rhoptry protein; TcTASV-C, T. cruzi Trypomastigote Alanine, Valine and Serine rich proteins; PFMSP1, P. falciparum merozoite surface protein*.

*Summary: Combining filtration/concentration and ultracentrifugation through sucrose gradient cushion retain intact membrane vesicles. Commercial exosome purification kit which could precipitate a wider or more restricted range of vesicles has also been used for PPEVs isolation but its validation requires categorical proof. Populations of vesicles obtained by differential centrifugation and ultracentrifugation, most often provides a mixed population of EVs (Colombo et al., 2014) and soluble proteins that are usually not associated with vesicles (Bayer-Santos et al., 2013). Size exclusion chromatography has been advocated in situation of intended higher yield of PPEVs (Nievas et al., 2018). In the grossest sense, the method adopted to isolate EVs has considerable effects on its proteomic profile and thus compounds the difficulty to extrapolate findings between different proteomic studies of PPEVs. More so, recovered EVs after filtration of culture may not give complete representation of parasites extracellular products. Aside this, physicochemical stimuli also play important roles in the content and function of isolated EVs*.

Also, EVs are classified on the basis of biochemical properties (Kowal et al., 2016) and membrane surface proteins (Wu et al., 2019) which are often used as EV markers (Théry et al., 2018). All categories of EVs have tetraspanins (CD63, CD81, CD82), major histocompatibility (MHC) 1, integrins, endosomal sorting complex required for transport (ESCRT) I-III, ALIX proteins, heath shock protein (HSP) 70, cytoskeletal proteins, and GAPDH as surface markers (Yanez-Mo' et al., 2015; Théry et al., 2018). Importantly, in eukaryotes, CD63, CD9, HSP 70, TSG101, flotillin, and Rab5b are common markers for exosomes (Shao et al., 2018; Gill et al., 2019), whereas microvesicles can be identified by selectins, annexin V, flotillin-2, and CD40, and apoptotic bodies distinctively express annexin V, DNA histone, phosphatidylserine, and genomic DNA as specific markers (Couper et al., 2010; Shao et al., 2018; Wu et al., 2019). Specific transmembrane proteins (e.g., epidermal growth factor receptors) and adhesion proteins (e.g., epithelial cell adhesion molecules) are important pathophysiological EV biomarkers (Shao et al., 2018). Correspondingly, pathogenic protozoa such as *Leishamania* spp have expressed cytoskeletal protein (e.g., actin and tubulin), HSP70, HSP90, HSP83/90, and elongation factor-1 α (EF-1α) as EV markers (Silverman et al., 2010a; Castelli et al., 2019) and many soluble proteins that are contained in the vesicles (Ribeiro et al., 2018). However, a large proportion of the components within microparticles are yet undefined. Again, in some instances, EV markers may not be significantly expressed as observed with annexin V of *P. berghei* (Couper et al., 2010).

Common factors involved in EV secretion especially ESCRT have been shown in the secretion of *T. brucei* exosomes in which the suppression of Vps36, an ESCRT component, led to the compromise of *T. brucei* exosome secretion (Eliaz et al., 2017). Before this finding, it was reported among *Leishmania* spp that, vesicle secretion is rather homologous to the classical exosome secretion pathway found in higher eukaryotes (Atayde et al., 2015). Despite the absence of typical MVBs, *Giardia lamblia* trophozoites exosome-like vesicles were formed in the endosome/lysosome peripheral vacuoles with the involvement of ESCRT, Rab and ceramide (Moyano et al., 2019). Distinct functions of ESCRT in the formation of PPEVs may include mobilization, docking, and fusion (Reiner et al., 1996). The secretion of vesicles when MVBs fuse with lysosomes is also possible in parasitic protozoa (de Souza and Barrias, 2017). In spite of these varying reports, secretion of vesicles by *L. brucei* supposedly occurred by active exocytosis (Geiger et al., 2010), whereas findings on *Giardia intestinalis* microvesicle secretion supported the involvement of cholesterol (Evans-Osses et al., 2017), but it is not yet clear if this phenomenon occurs in all pathogenic protozoa.

In *Plasmodium*, some deviations in the formative process of EVs have been observed. Kehrer et al. (2016) reported that the changes in *P. berghei* exocytic inner membrane compartment led to the eventual fusion of the exocytic membrane and the parasite plasma membrane with subsequent formation of exosome-like structures. Another way of secretion of EVs in *P. berghei* is the reported selective clearance or degradation of some sporozoite organelles with temporal and spatial regulation of membrane components which are finally sorted and packed into vesicles (Jayabalasingham et al., 2010). Consequently, microvesicles from *P. falciparum*-infected red blood cells (RMVs) are distinct from post-rupture vesicles released before parasite egress from red blood cells (Mantel et al., 2013).

Vesicle formations by the budding process from the flagellar pocket are common with species of *Leishmania* and Trypanosomes. Additionally, all developmental stages of *Trypanosoma cruzi* and *T. brucei* perhaps have EVs bud off from the plasma membrane (Torrecilhas et al., 2012; Szempruch et al., 2016b). Incidentally, exosomes or microvesicle in *T. cruzi* conceivably have their origin from the tubular network of the endoplasmic reticulum and Golgi (Lozano et al., 2017). Also, there could be plasma membrane-derived vesicles and exosomes formed through fusion of MVBs with the flagellar pocket of epimastigote and metacyclic stages of *T. cruzi* (Bayer-Santos et al., 2013), whereas *Trichomonas vaginalis* microvesicles are derived from endocytic compartments or the plasma membrane (Rada et al., 2019).

Bizarre forms of *T. brucei* vesicles which are independent of ESCRT machinery and autophagy have been observed in addition to EV exocytosis from the flagellar pocket, parasite surface, and MVBs (Eliaz et al., 2017). *Leishmania* spp, on the contrary, use predominantly non-classical mechanisms to direct the release of microvesicles, exosome-like vesicles, apoptotic vesicles, and glycosomes (Silverman et al., 2008). *Giardia lamblia* bulbous excretory secretory vesicles (ESVs) were formed as clefts directly from the early dilation of rough endoplasmic reticulum cisternae (Lanfredi-Rangel et al., 2003). Apparently, the biogenesis and origination of PPEVs seem to be peculiar to parasitic protozoan species and en route differently from the parasite subcellular compartments (Table 1).

Pathogenic protozoa, at different developmental stages, can secrete mixed population of exosomes, microparticles (MPs), and apoptotic bodies (Garcia-Silva et al., 2014; Siedlar et al., 2017). Isolates of EVs from similar, but clinically divergent, species of *Leishamania* have displayed distinct profiles (Silverman et al., 2010a). Such distinct EV profiles depend on the life stage, strain, and population of *T. vaginalis* (Twu et al., 2013), *P. falciparum* (Regev-Rudzki et al., 2013), and *T. cruzi* (Moreira et al., 2019). Nevertheless, parasite-shed vesicles are an additional general mechanism that is central to parasite pathogenicity (Torrecilhas et al., 2009). Several specific terms for PPEVs are listed in Table 1.

## Stressors for PPEV Secretion

A large number of parasite niches in hosts and environmental factors are known to orchestrate the release of EVs (Torró et al., 2018). The complexities surrounding the secretion of PPEVs are due to diverse biochemical, physical, and mechanical stressors directed against the parasites *in vivo*. The secretion of PPEVs can be initiated by developmental changes in the parasite's life history as observed in *P. falciparum*, where developmental transition from the trophozoite, schizonts and the ring stages led to increasing MVs secretion (Barteneva et al., 2013). Human serum, at 10-fold bile concentration and pH 3 and 8, has been used to induce microvesicles (MVs) in *G. intestinalis* trophozoites (Deolindo et al., 2013). *Entamoeba histolytica* trophozoite EVs were secreted in liver lesion (Chavez-Munguia et al., 2004) feasibly after parasite exposure to varying physiological and physical conditions in the gut of mice. Physicochemical stressors in the vector mid-gut can also orchestrate the secretion of *T. cruzi*s-derived EVs (Fernandez-Calero et al., 2015), and as reported by Gonçalves et al. (2018), *A. castellanii* EV secretion was triggered after re-cultivation in media without a protein source to mimic the physiological stress in the host's aqueous and vitreous humours.

Increasing parasite density could initiate the secretion of peculiar *P. falciparum* EVs *in vitro* (Correa et al., 2019). Congruently, nutrient-starved cultures are often being used to trigger EV secretion (Table 1) with claims that it mimics the hostile environment of the vector hind gut in the case of *T. cruzi* (Fernandez-Calero et al., 2015). On this premise, incubation of *T. cruzi* epimastigotes in culture media without fetal bovine serum (FBS) also triggered parasite transformation and disposal of some proteins via vesicle secretion (Bayer-Santos et al., 2013; de Souza and Barrias, 2017). However, chemical compositions of culture media will produce specific cargo that reflect the culture conditions and developmental stages of *T. cruzi* strains (Fernandez-Calero et al., 2015) just as comparable nutritional stress media triggered the release of different sizes of EVs among *T. cruzi* strains (Ribeiro et al., 2018). Apparently, serum starved media/chemically defined culture will trigger the formation of vesicles and induce apoptosis (Pope et al., 2013; Gonçalves et al., 2018) which may lead to myriad composition, size, and biosynthesis of PPEVs (Table 1).

Likewise, disintegration of *T. vaginalis* during *in vitro* cultivation produced vesicles which subsequently bind to the cell surface (Rada et al., 2019) and add to the existing subpopulations of EVs. The use of antibiotics as component of culture media may equally trigger typical secretion of EVs, although the implication of this was not clearly mentioned with the use of gentamicin as a component of culture media in the studies of *P. falciparum* EVs (Mantel et al., 2016; Sisquella et al., 2017; Castelli et al., 2019). Certainly, the biology of a specific parasite may play important roles in the preparation of culture media but it would be interesting to find out, if any, the functional difference (s) between PPEVs that are stimulated by drug and serum starvation and, also, the roles of drug-triggered EVs in parasite pathobiology in relation to drug selection and drug resistance because the involvement of HSPs in drug resistance among *Leishmania* strains has been speculated (Patino et al., 2019).

PPEV secretions have also been stimulated in culture media supplemented with calcium compounds (Cocucci et al., 2009). In fact, incubation of *T. vaginalis* with calcium chloride in regular media produced a 9-fold increase in parasite MVs (Nievas et al., 2018). The secretion of EVs by *G. lamblia* trophozoites has been recently shown to be stimulated by the addition of calcium into the culture medium (Moyano et al., 2019). Sodium compound at different concentrations and pH values have been shown to induce plasma membrane vesiculation in *T. cruzi* epimastigote (Da Silveira et al., 1979). Remarkably, purified tachyzoites of *Toxoplasma gondii* maintained in PBS at 37°C were able to secrete vesicles (Ramírez-Flores et al., 2019), but from the report of Barbosa et al. (2018), a temperature of 37°C impaired the secretion, biological effects, and cargo content of EVs released by *L. amazonensis* promastigote in culture.

Considerably high temperature at acidic pH induced the secretion of exosomes in *L. donovani/L. major* (Silverman et al., 2010b) and activation of *L. donovani* promastigote exosomes in parasitophorous vacuole (Deolindo et al., 2013), but only pH was responsible for *in vitro* secretion of the *T. cruzi* vesicle (Da Silveira et al., 1979). Meanwhile, massive secretion of exosomes by *Trypanosoma brucei* subjected to heat shock has been established (Eliaz et al., 2017). Conversely, Deolindo et al. (2013) hypothesized that *Giardia intestinalis* trophozoites released MVs to resist pH change. There is also a report of exosome secretion by co-incubation of genetically modified and wild-type strains of *T. brucei*, but changes in environment factors prominently influenced the release of vesicle and cargo content (Torrecilhas et al., 2012). Likewise, the formation of vesicles by pathogenic protozoa can also occur in responses to host cell interaction (Nievas et al., 2018). The concern would be to find out if the degree of parasite virulence corresponds to vesicle secretion.

Relatively, centrifugation steps can disorganize tubules into vesicles similar to ectosomes and exosomes (Ramírez-Flores et al., 2019), indicating that laboratory treatment may influence extant populations of EVs, but whether tubule disorganization into the vesicle by gravitational shearing represents EV activation through cell–cell abrasions or cell–parasite contacts requires further clarification. Nonetheless, other stressors that can initiate the secretion of PPEVs include cell topography, apoptosis or autophagy (Yanez-Mo' et al., 2015), hypoxia, and irradiation (Torró et al., 2018). Substantial secretion of EVs by parasitic protozoa is due to nutritional stress, pathogenesis, anti-proteolysis, antigen presentation, and parasite growth. The consideration to find combinatory stressors that support the maximum secretion of EVs and the physiological/behavioral implications for a specific protozoan parasite will be good for the field. Nonetheless, *Acantamoeba castellanii* EVs could equally act as stressors and induce the secretion of EVs from the host cell membrane (Gonçalves et al., 2018).

## Functions of PPEVs

The biological functions and cargo composition of PPEVs are dependent on the parasite from which they are secreted (Deolindo et al., 2013), and the amount of EVs secreted by *L. amazonensis* promatigote is dependent on the period of exposure to stressors (Barbosa et al., 2018).

### Roles of PPEVs in the Parasite Community

The formation and release of vesicles enhance survival, transmission, and mitotic multiplication of pathogenic protozoa (Roditi, 2016). Also, vesicle-dependent and proportional 5-fold increase in absolute number of *T. cruzi* trypomastigote suggests that parasite-derived vesicles could initiate parasite replication (Garcia-Silva et al., 2014). Conversely, *P. falciparum-*infected cell*-*secreted EVs carried suicidal signals that could induce parasite death (Correa et al., 2019). Moreover, this raises the question: are PPEVs carrying death signals directed against other parasites in the population, or are they self-targeting?

PPEVs can actuate intra- and inter-specific quorum sensing. For instance, *T. vaginalis* vesicles interacted with other *T. vaginalis* strains and, in the process, enhanced cyto-adherence of the recipient strain (Nievas et al., 2018), which showcases EVs as mediators of intra-specific interactions (Twu et al., 2013). Likewise, tachyzoites of *T. gondii* invaded more cells when incubated with *T. cruzi* EVs. However, the incubation of *T. gondii* and EVs from *Crithidia mellificae* choanomastigotes did not orchestrate an increase in the number of infected cells (Moreira et al., 2019). Even high concentrations of MVs from *G. intestinalis* were unable to enhance the invasion of *T. cruzi* metacyclic trypomastigotes (Evans-Osses et al., 2017). Taken together, these evidences suggest that EVs can enhance parasite pathogenesis by exposing certain host cell surface ligands or molecules which the invading parasites can recognize, but how protozoan parasites distinguish EVs from unrelated species is yet to be determined.

EVs also play important roles as channels of intercellular communication within parasite population (Gavinho et al., 2018). Additionally, as suggested by Geiger et al. (2010), *T. brucei* may possibly use MVs to communicate between trypanosomes by exchanging non-protein cytosolic receptors or genomic information to harness survival strategies. Similarly, the vesicles of *T. vaginalis* might have functional roles in mediating communication between parasites during infection (Nievas et al., 2018). Intact exosomes can potentially regulate parasite migration by transmitting repulsive signals to facilitate communication and social migration of *T. brucei* (Eliaz et al., 2017). Secretory vesicles have likewise been shown to affect parasite motility and egress from infected cells. Specifically, certain G377-containing vesicles enhance parasite egress from parasitophorous vacuoles and RBC membranes. Inability of *P. berghei* to secrete such vesicles obliterated further transmission and motility (Kehrer et al., 2016). RMVs from *Plasmodium*-infected RBCs could as well stimulate and regulate gametocyte production (Mantel et al., 2013).

### PPEVs in Host-Parasite Interactions

**Figure 1:**
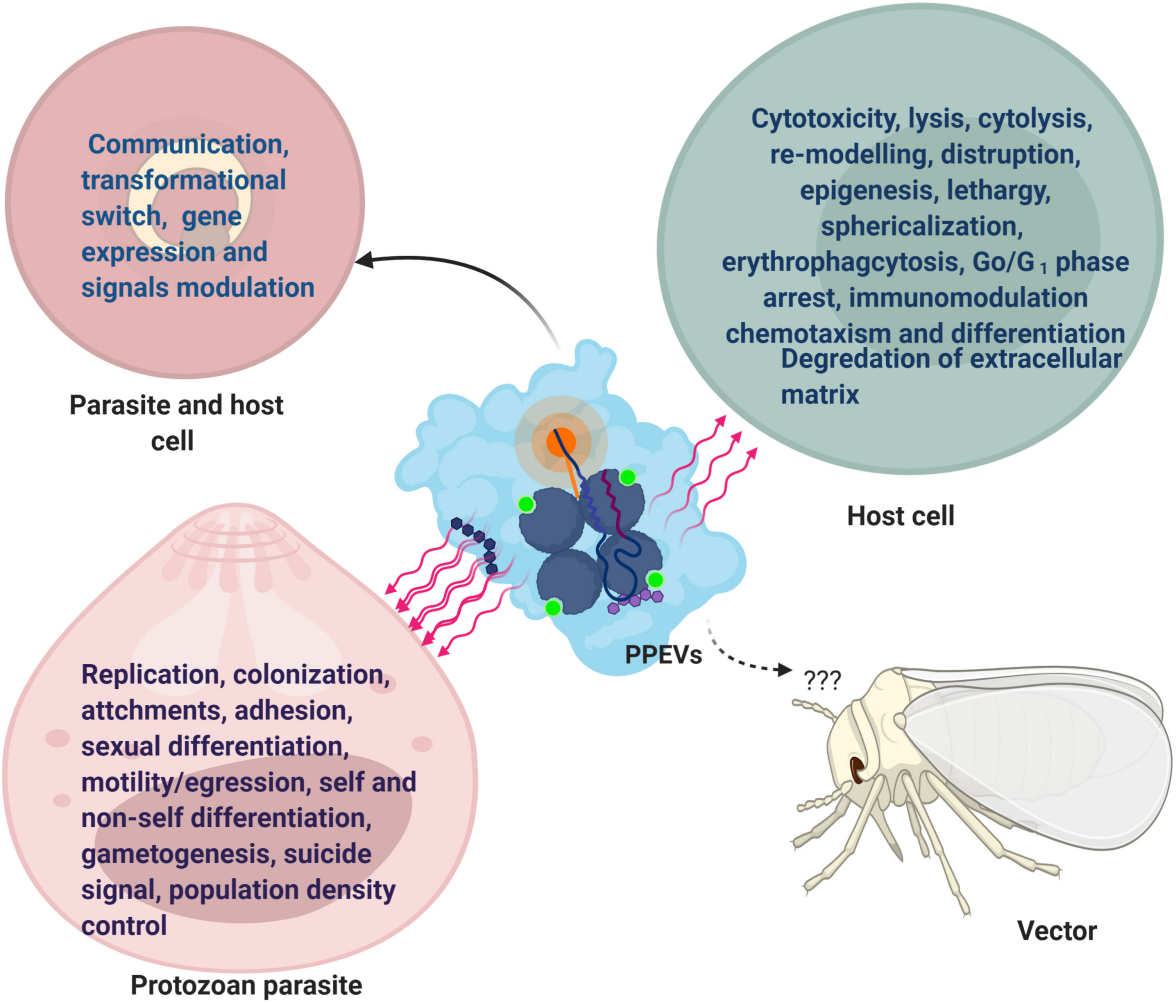
Functional Effects of PPEVs. After-effects of PPEV interaction and internalization by recipient cells and protozoan parasites. The common effects in parasites and host cells are seen in communication, development, and gene expression.

Szempruch et al. (2016a) suggested that communication between host cells and *T. brucei* occurred via assemblage of its fusogenic EVs which may serve as vehicles for parasite-host cell transfer of membrane proteins as *L. donovani* exosomes showed long-range communication with naive host cells (Silverman et al., 2010a). EVs derived from *P. falciparum*-iRBCs traverse the infected cells and are capable of promoting a parasite developmental switch (Mantel et al., 2016). Remarkably, exosome-like vesicles of *P. falciparum*-iRBCs transferred parasite DNA and acted as an emissary that induced sexual differentiation, parasite survival, and communication within the population of parasite-infected red blood cells (iRBCs) (Regev-Rudzki et al., 2013). *in vitro* incubation of EVs containing DNA with host cells revealed higher mRNA induction in host cells and is suggestive of the fact that PPEVs are carriers of signal molecules and could travel farther in cytosolic milieu (Sisquella et al., 2017) (Figure 1).

Shed vesicles represent an additional mechanism by which parasites present antigens to the host and play a pivotal role in acute parasitic disease. *T. cruzi* vesicles interact with target cells in ways that may be difficult for free molecules, and as such, exhibit the horizontal transfer of parasitic molecules and parasite extensions (Yanez-Mo' et al., 2015). Similarly, in the context of default survival plan by protozoan parasites, the release of EVs may be an efficient strategy employed by the parasite to protect parasitic biomolecules against extracellular degradation (Bayer-Santos et al., 2013). This is in accordance with the general function of MVBs to prevent cells from proteotoxicity through the formation and accumulation of intraluminal vesicles (ILVs) (Lozano et al., 2017). Also, co-egested *L. major* exosomes with *Leishmania* parasites during a blood meal by an infected sand fly possibly exert separate influence during transmission and early events of an infectious process in the host (Atayde et al., 2015), but the effects of parasite-derived EVs on vectors have not been reported (Figure 1).

EVs have the potential to increase parasitemia in host. For instance, prior inoculation of *T. cruzi-*derived EVs in mice showed over two times the number of parasites in blood and two times more amastigote nests in hearts (Lovo-Martins et al., 2018). The inoculation of *L. infantum* extracellular products potentiates dose-dependent infection *in vivo*, and EV populations significantly correlated with parasite numbers (Pérez-Cabezas et al., 2018). The role of EVs in host cell invasion is typified by the ability of *T. vaginalis* exosomes to prime the urogenital tract for the purpose of parasite colonization (Twu et al., 2013). Further still, the addition of *G. intestinalis* MVs to methyl-β-cyclodextrin-mitigated *G. intestinalis* trophozoites restored its attaching ability for subsequent invasion (Evans-Osses et al., 2017).

Moreover, the prior inoculation of *T. cruzi* vesicles in mice before parasite infection heightened pathogenicity to 100% mortality. These *T. cruzi*-shed membrane vesicles aggravated severe heart inflammation and increased the number of intracellular amastigote nests. It was shown further that *T. cruzi* vesicles could not directly induce significant pathology in mice, and injection of *T. cruzi* EVs into mice before trypomastigote infection led to a transient but substantial increase in parasitemia (Torrecilhas et al., 2009). *L. major* exosomes possibly heightened the formation of a footpad lesion in mice as co-inoculation of *Leishmania* parasite and *Leishmania* exosomes exhibited a 3–4-fold increase in lesion volume than with parasites alone (Atayde et al., 2015). Barbosa et al. (2018) reported similar footpad lesion due to *L. amazonensis* EVs as well as increased parasitic load. Unassumingly, *T. cruzi-*derived vesicles may influence parasite proteolytic activity on the host tissue (Torrecilhas et al., 2009). However, the lytic effect of purified *T*. *cruzi* vesicles on host cells was transiently local to the site of inoculation in mice, suggesting that not all the RBCs in the circulation are equally vulnerable or probably lack certain corresponding molecular signatures (Roditi, 2016).

Perceptibly, initial host cell treatment with PPEVs would increase host cell parasitization. For instance, EVs of *T. cruzi* Y strain made the host cell more susceptible to parasite entry in the first moments of infection (Lovo-Martins et al., 2018). Dong et al. (2019) have lately reported that EVs have the capacity to favor infection and propagation of parasites in the hosts. Besides the fact that secreted vesicles have ability to fuse with susceptible host cells, parasite-derived MVs can fuse with host cell-derived MVs (Ramirez et al., 2017), and the amount of reacting EVs is proportional to the percentage of infected cells (Ribeiro et al., 2018). Such level of host-parasite interaction needs further elucidation. Furthermore, *Acanthoamoeba catellanii* EVs have shown cytotoxic effects and hampered mammalian epithelial cell viability by the action of its degrading enzymes. However, the extent of host cell damage was dependent on cell type and the dose of *A. castellani* EVs (Gonçalves et al., 2018) (Figure 1).

Similarly, there is report of cell sphericalization, disruption, reduction in adhesive ability, and cytolysis of rat glial cells following the exposure to *A. castellanii* EVs (Lin et al., 2019), just as *T. gondi* exosomes affected macrophage viability in dose-dependent mode (Li et al., 2018a) and EVs secreted by *T. cruzi* induced epigenetic changes in susceptible mammalian cells (Fernandez-Calero et al., 2015). When *T. brucei* EVs were fused with mammalian erythrocytes, erythrophagocytosis, the cause of anemia during acute trypanosomiasis, was reported (Szempruch et al., 2016a). In Chagas disease, *T. cruzi* EVs and the incorporated mucin and mucin-associated surface protein (MASP)-specific peptide inhibited host cell lysis facilitated by the human complement system (Lozano et al., 2017). Apart from reported pathological fall out, EVs from *P. falciparum-*iRBCs contributed to vascular dysfunction, endothelial activation/leakage, and parasite sequestration (Mantel et al., 2016) (Figure 1).

Experimental evidence suggests that shed vesicles of the *T. cruzi* sE48 strain significantly enhanced metacyclogenesis of the host cell (Garcia-Silva et al., 2014). Also, the incubation of EVs of the *T. cruzi* Pan4 strain with Vero cells induced intracellular mobilization of Ca^2+^, causing the reversible disruption of the actin filaments and formation of filopodia, and finally halted cell cycle at G0/G1. Promastigote and amastigote exosomes of *L. infantum* caused host cell chemotaxism, and *L. infantum* amastigote exosomes specifically caused the differentiation of monocytes into macrophages (Castelli et al., 2019). Similarly, Moreira et al. (2019) demonstrated that *T. cruzi*-derived EVs could alter host cell architecture, membrane permeabilization, and exposure of epitopes to antibodies (Figure 1).

Nonetheless, the thermal treatment of *T. cruzi* EVs and enzymatic/chemical treatment with proteases and sodium periodate inhibited *in vitro* host cell parasitization by trypomastigotes of *T. cruzi* (Moreira et al., 2019). Moreover, disruption of the exosomal membrane and boiling abrogated the *L. major* exosome ability to enhance lesion size and decreased parasite load, suggesting the fact that intact exosome integrity plays important roles in diseases progression (Atayde et al., 2015). An example of a PPEV effect outside the host cell is the report of liberated electron dense granules proposed to be contained in vesicles of *E. histolytica* trophozoites, which could initiate the degradation of extracellular matrix through the action of its proteolytic enzymes (Chavez-Munguia et al., 2004) (Figure 1). It is not clear, however, whether there are degredative (lytic) and messenger PPEVs that are used to target host cells and protozoan parasites, respectively.

## Mechanisms of PPEVs Internalization

EVs have different half-life because they can quickly be taken up by target cells and thus exist only around the pathogen (Théry et al., 2009). Also, EV disappearance from circulation may be due to its uptake during interaction with the target cell and in the process becomes internalized (Mantel et al., 2016; Eichenberger et al., 2018). The disappearance of *Leishmania* exosomes is likely due to cellular uptake, membrane dissolution, or subsequent degradation after binding to naive cells (Silverman et al., 2010a). The approximate time for PPEV existence in circulation before cellular uptake is exemplified by *T. cruzi*-shed membranes that had a half-life of 3.5 h with respect to the half-life of Tc-85 protein released by the parasite (Torrecilhas et al., 2009). This is comparably consistent with other experimental evidence that posited 3 h as enough time for exosome uptake by the target cell (Cheng and Zeng, 2019). However, pathogenic protozoan exosomes have been hypothesized to have different kinetics in target cells (Silverman et al., 2010a).

### PPEVs Internalization by Host Cells

**Figure 2:**
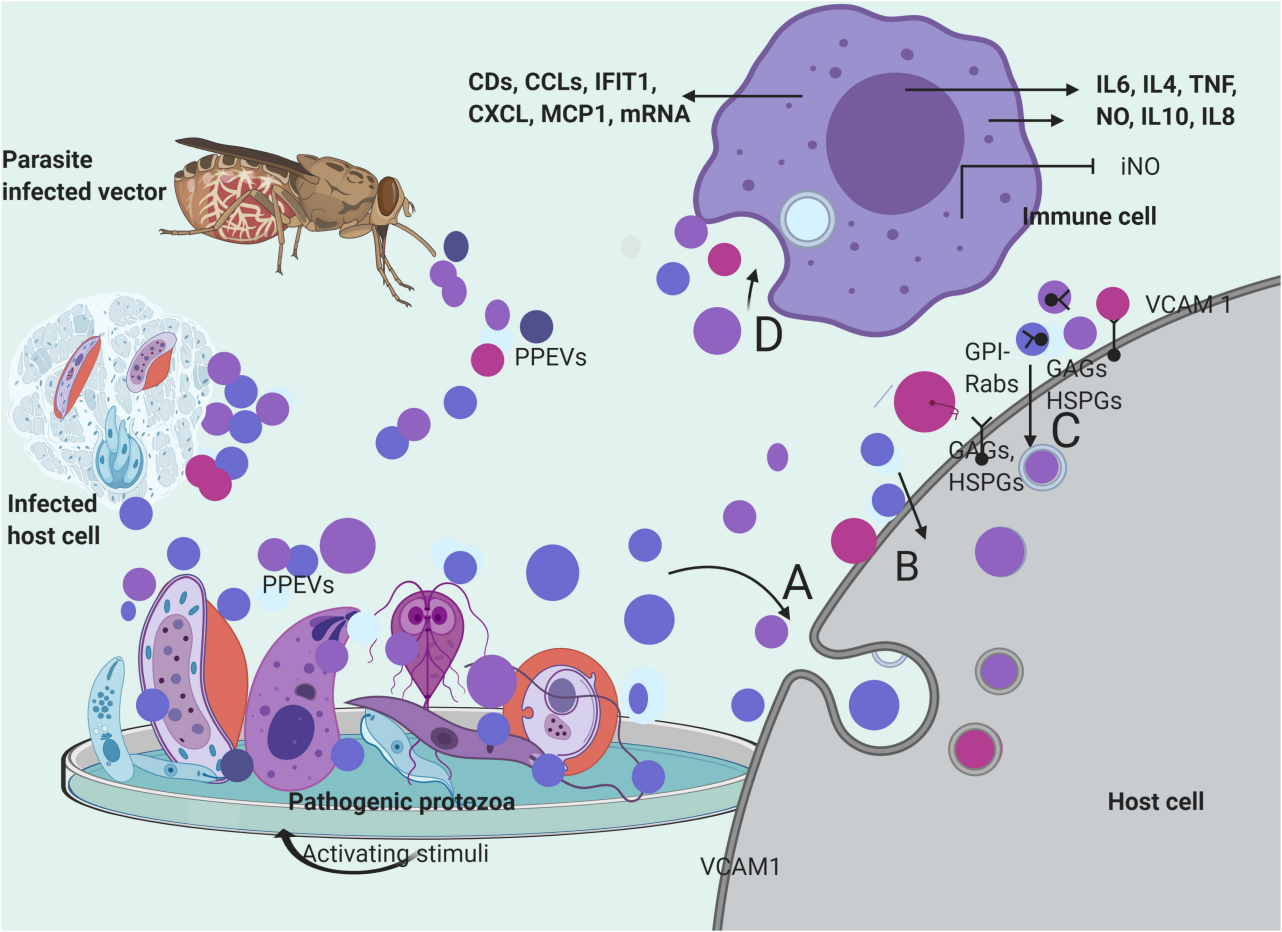
Secretion and mechanisms of PPEVs internalization. PPEVs are formed after protozoan parasite exposure to various chemical and mechanical triggers in host cells, *in vitro*, and in the gut of vectors. After secretion, PPEVs are quickly taken up by susceptible cells, but various mechanisms have been proposed for internalization process. **(A)** bulk transport of PPEVs across the cell membrane via endocytic assimilation involving phagocytosis and pinocytosis. **(B)** PPEVs fuse with the host target cell before consequent internalization which may be mediated by Caveolin-dependent pathways. Lipid rafts, cholesterol, and lectin on PPEV membranes and host cells play significant roles in this respect. In the process of caveolin-dependent pathway, host cell caveolin-1 acts as regulator, and HSPGs acts as receptors for *T. vaginalis* EVs. Alternatively, using the mechanisms of membrane transporters, *L. donovani* exosomes could hijack the host retrograde trafficking pathway to directly dump exosomal cargo into the host cell (Garcia-Silva et al., 2014). **(C)** Possible uptake of PPEVs via receptor-ligand interactions involving receptors and proteins on host cells and membranes of PPEVs. Clathrin is a protein scaffolding found coating eukaryotic vesicles, which plays important role in receptor-mediated endocytosis of PPEVs through the plasma membrane associated with different adaptor proteins for clathrin-coated EVs. PPEV membrane protein can thus interact with receptors on the target cell and activate intracellular signaling. In this process, Rab5 protein mediates endocytosis and fusion of clathrin-coated vesicles. Also, GPI anchors on the vesicles may facilitate fusion to the host cell. Surface expression of vascular cell adhesion protein-1 (VCAM1) in endothelial cells has been demonstrated as a host cell response to iRBC EV uptake, which is significant in vascular dysfunction (Mantel et al., 2016). **(D)** Process by which immune cells interiorize PPEVs. A fundamental basis for PPEV internalization by immune cells has been linked to endocytosis and phagocytosis. There has been no empirical proof for ligand-receptor-mediated fusion and internalization of PPEVs by host immune cells. However, internalization of PPEVs by immune cells can redirect cytokine secretions and differential regulation of immune pathways.

There are indications that a large number of protozoan parasite exosomes can be internalized by host cells (Li et al., 2018a) with postulated mechanisms of receptor-mediated, fluid-phase endocytosis, or direct fusion with host cells (Szempruch et al., 2016b). *T. cruzi*-derived vesicles have been suggested to be endocytosed by host cells (Bayer-Santos et al., 2013). Hypothetically, vesicular content can be delivered to host cells through fusion of EVs with the host cell, endocytotic assimilation, or progressively by a control-delivery system (Roditi, 2016; Li et al., 2018b). The sequence of PPEV internalization might involve binding to host cells through receptor–ligand interactions, and in the process become putatively attached to the target-cell membranes before endocytosis (Valadi et al., 2007). Ramírez-Flores et al. (2019) reported that endosome-associated Rab proteins played some roles in the fusion of *L. infantum* vesicles to host cells and the formation of tubules even though electroporation of myelogenous leukemia cells with *T. cruzi* epimastigote EVs showed clear incorporation of labeled EVs following a series of endocytosis and exocytosis (Garcia-Silva et al., 2014) (Figure 2).

Glycosaminoglycans (GAGs), heparan sulfate proteoglycan (HSPGs) chains, and other unidentified host cell surface components mediated *T. vaginalis* EV uptake because the loss of host cell surface GAGs and HSPGs reportedly reduced, but did not completely block the uptake of *T. vaginalis* EVs (Rai and Johnson, 2019). Furthermore, the treatment of the host cells with a specific inhibitor of lipid raft-mediated endocytosis reduced *T. vaginalis* EV uptake to a considerable point. To this end, the cellular uptake of *T. vaginalis* EVs required cholesterol in addition to caveolin-dependent, lipid raft proteoglycan-mediated endocytosis (Rai and Johnson, 2019). Incidentally, Garcia-Silva et al. (2014) reported *T. cruzi* vesicles coated with clathrin from early endocytosis and the trans-Golgi membrane network. However, the inhibition of both Clathrin-mediated and caveolin-dependent endocytosis through Dynasore blocked EV uptake. This substantiates the endocytic internalization of *P. falciparum*-iRBC-derived EVs by endothelial cells (Mantel et al., 2016) and phagocytosis-like mechanisms through which *P. falciparum*-iRBC-derived microvesicles (RMVs) were enclosed in additional membranes after internalization (Mantel et al., 2013) (Figure 2).

Moreira et al. (2019) reported that *T. cruzi* EVs appear to adhere to the host cell through its surface lectin scaffolds, but the presumed enzymatic activities of glycosylated proteins of *T. cruzi* EVs during adhesion/internalization by the host cell require elucidation. Also, fusion with the host cell, before cargo delivery, has been reported in the case of *T. vaginalis* exosomes (Twu et al., 2013). *A. castellani* EVs were first found localized and accumulated within the phospholipid-rich membrane of epithelial cells before cytoplasmic phagocytosis. The elapsed accumulation of the amoebic EVs within the lipid epithelial membrane indicates the involvement of host cell lipid raft in the internalization process (Gonçalves et al., 2018) (Figure 2). However, the physiological mechanism that produced epithelial cell membrane associated EVs after *A. catellani* EV adhesion requires further elucidation. Similarly, Rat glial cells have internalized *A. castellanii* EVs but the mechanism involved remains unknown (Lin et al., 2019) (Figure 2).

In respect to PPEV internalization by immune cells, stained EVs of *Neospora caninum* have been found to randomly accumulate within the cytoplasm of macrophages (Li et al., 2018c), while fluorescent *P. falciparum*-iRBC-derived EVs were observed in the perinuclear region of the human bone marrow-derived endothelial cell (Mantel et al., 2016). Importantly, temperature could have facilitated this endocytotic internalization because *P. falciparum*-iRBC-derived EVs were significantly detected in monocytes at 37°C (Sisquella et al., 2017), as well as *G. intestinalis* MV internalization by immature dendritic cells (iDCs) which were later inhibited almost completely at 4°C and by the addition of cytochalasin D (Evans-Osses et al., 2017). In addition, iRBC-derived EV internalization by macrophages was sensitive to cytochalasin D (Mantel et al., 2016) (Figure 2). However, apart from the probable endocytotic process, it is yet to be determined if dyes confer additional properties on PPEVs to facilitate internalization by immune cells. In summary, the peculiarity of the host cell, plasma membrane architecture, PPEV lipid membrane, and cargo content plays significant roles during the internalization of protozoan parasite EVs (Figure 2).

### PPEV Internalization by Protozoan Parasites

Studies have shown that protozoan parasites can as well-internalize EVs from related and unrelated species. *T. cruzi*, speculatively, makes use of a clathrin-mediated endocytosis machinery to internalize the tsRNA cargo of exogenous sources (Garcia-Silva et al., 2014), whereas, endocytic activity in *T. brucei* correlated with expression levels of the clathrin-independent pathway due to the enrichment of GPI-anchored proteins on the *T. bricei* plasma membrane (Allen et al., 2003) which is also found enriched in EVs (Raposo and Stoorvogel, 2013). It can be deduced, therefore, that the use of both autonomous clathrin-mediated endocytosis and clathrin-independent pathway (via GPI-anchors) may explain the short half-life and rapid disappearance of PPEVs. The incubation of exosomes from modified *T. brucei* with *T. brucei* wild-type revealed that the co-opted *T. brucei* exosomes were observed around or within the lysosomes of *T. brucei* wild-type (Eliaz et al., 2017).

Protozoan parasites may use contiguous receptor-independent endocytosis to internalize vesicles despite the array of protein anchors and membrane receptors on protozoan parasites. To substantiate this, Szempruch et al. (2016b) reported that the binding and uptake of *T. brucei* EVs by *T. b. brucei* was receptor-independent, which was first mediated by fusion with the mammalian erythrocyte membrane. Also, PKH67-labeled RMVs have been efficiently incorporated into *P. falciparum*-iRBCs with eventual accumulation in the parasite nuclear periphery, but only a subset of RMVs were internalized, signifying that not all *P. falciparum-*iRBCs were receptive to RMV uptake (Mantel et al., 2013). In congruence with EV fusion through lipid raft, EVs from *T. b. brucei*^*SAR*−*Ty*^ fused with the membrane and flagellar pocket of adjacent *T. b. brucei*, which led to the internalization of the vesicles and associated protein in the endolysosome (Szempruch et al., 2016b). Furthermore, the internalization of EVs by parasitic protozoa can be aided by dissolution of parasite plasma membrane. For example, detergent treatment of *T. b. brucei* increased its membrane permeabilization and ensued the transfer of serum resistance-associated (SRA) proteins contained in *T. b. brucei* EVs (Szempruch et al., 2016b).

## PPEVs Bioactive Molecules: Exports and Functions

Among parasitic protozoa, the composition of EVs includes proteins, carbohydrates, lipids, nucleic acids, virulence factors, resistant genes (Szempruch et al., 2016b; Sisquella et al., 2017), and unprocessed proteins (Lozano et al., 2017). Also, some PPEV-encapsulated biomolecules are classical EV markers (Mantel et al., 2013), immune modulators, mediators of intracellular signaling, host-parasite interactions, membrane fusion, transporters, and oxidation-reduction processes (Geiger et al., 2010; Moreira et al., 2019). However, the functions of PPEVs rely on preparation, time of reaction, temperature, pH and most importantly, species and strain of origin (Twu et al., 2013; Montaner et al., 2014; Nogueira et al., 2015; Silva et al., 2018; Moreira et al., 2019) (Table 1). PPEV bioactive molecules immensely contribute to parasite development, and it is likely that protozoan parasites secrete biochemically different EVs at every developmental stage so as to adapt to a changing environment as exemplified by *L. infatum* which has significant enrichment of ribosomal and RNA transport proteins during the parasite growth at the log phase but an abundance of cellular processes and oxidative phosphorylation proteins at the stationary phase (Santarém et al., 2013).

### Nucleic Acids

The secreted nucleic acids in PPEVs vary with organisms, activating factors, and the subgroups of the EV population. Distinct types of small RNAs (sRNAs) in *A. castellanii* EVs were reported to be modulated by nutritional stress (Gonçalves et al., 2018). In addition, *T. cruzi* epimastigotes under nutritional stress have a specific population of sRNA packaged into their vesicles for possible interactions with host cells (Fernandez-Calero et al., 2015). PPEVs are carriers of messenger RNAs (mRNAs), microRNAs (miRNAs), different types of mediators (de Souza and Barrias, 2017), RNA, and genomic DNA (gDNA) (Sisquella et al., 2017). Functionally, mRNAs in *T. gondii* EVs were recognized by the host immune system (Silva et al., 2018), and *T. cruzi*-secreted EVs contained sRNA, transfer RNA (tRNA), (small nucleolar) sno/(small nuclear) snRNAs, and specific Piwi proteins in complex association with ribonucleoprotein (Fernandez-Calero et al., 2015). The differential packing of sRNAs in PPEVs has revealed that the distinction between *Leishmania* epimastigote and metacyclic trypomastigote stages (Bayer-Santos et al., 2014). Garcia-Silva et al. (2014) demonstrated the release of nucleic acids from PPEVs by showing that tsRNAs in *T. cruzi* vesicles are delivered to adjacent *T. cruzi*. Mantel et al. (2016) reported that tsRNAs of *T. cruzi* epimastigote MVs have a longer nucleotide sequence than tsRNAs in the *T. cruzi* subcellular region. Conversely, secreted exosomes of *T. brucei* containing spliced leader RNA (SL RNA) affected the social motility of procyclic trypanosome with sheer dependence on perceptible genetic signal (Eliaz et al., 2017), and *P. falciparum*-derived vesicles altered cellular function via changes in EV-derived miR-451a-Argonaute2 complexes and target gene expressions (Mantel et al., 2016; Rivkin et al., 2017). Nevertheless, the potential functions of abundantly detected non-coding RNAs in the *P. falciparum-*EVs are yet to be clarified (Sisquella et al., 2017) as well as the extra nucleotide extension of tsRNAs of *T. cruzi* MVs.

### Proteins and Virulence Factors

The secreted exosomes of *L. major* promastigotes and amastigotes function as the main protein secretory pathway (Leitherer et al., 2017), and the involvement of *T. vaginalis* vesicles in the export of adhesin proteins has been confirmed by immunofluorescence analyses (Rada et al., 2019). Of the total proteins released by *T. cruzi* EVs, prediction holds that about 57% were secreted through classical and non-classical pathways. This therefore lends credence to the evidence that the *T. cruzi* secretome is formed by proteins that are transported in EVs (Lozano et al., 2017). The proteomic analysis of *T. brucei*-derived EVs speculated that the flagellum might play a considerable role in the sorting and delivery of its biologically active molecules to neighboring cells (Szempruch et al., 2016b). *T. gondii* tachyzoite vesicles contained dense granular protein with an indication that dense granules and self-assembled vesicle-tubular structures are a potential source of proteins in the vesicle (Ramírez-Flores et al., 2019). Essentially, properties of *L. donovani* wild-type and mutant strain vesicles were influenced by the specificities of cargo packaging regulated by HSP100 (Silverman et al., 2010b). Additionally, the putative pantothenate protein (PAT) and HSP100 of *P. berghei* secretory vesicles were necessary for the expulsion of vesicular content into the parasitophorous vacuole (Kehrer et al., 2016), just as extracellular *T. gondii* tachyzoites constitutively secreted soluble components of the vesicles within the parasitophorous vacuole (Ramírez-Flores et al., 2019). Meanwhile, a subtle contrast has been found in *T. cruzi* where the predominant EV proteins were likely anchored on the parasite surface via GPI lipid or inserted into the EV membrane past a conserved C-terminal region (Bautista-lópez et al., 2017).

The functional array of molecules in pathogen-derived EVs has been concisely reviewed by Kuipers et al. (2018) (Table 1). Succinctly, EVs from *A. castellanii* are purportedly rich in aminopeptidase and proteases which contributed to the pathogenesis, host tissue damage, and cell death (Gonçalves et al., 2018; Lin et al., 2019). Enteric *Entamoeba histolytica* has also been reported to secrete vesicles containing actin and cationic proteins with proteolytic activities (Chavez-Munguia et al., 2004). As well, *T. vaginalis* exosomes contained surface proteins and proteases which enhanced parasite adherence (Twu et al., 2013), though the presence of proteases, kinases, and glycosidases in *A. castellani* EVs contributed to parasite establishment and the colonization of the host tissues (Gonçalves et al., 2018). *P. falciparum* lactate dehydrogenase with relative abundance in *P. falciparum*-iRBC-derived EVs had the capacity to communicate a suicidal signal (Correa et al., 2019).

Fundamentally, a large proportion of proteins and virulence factors are secreted in membrane-bound vesicles (Deolindo et al., 2013; Ribeiro et al., 2018), but at this point, it is needful to point out that the expression of virulence factors in PPEV cargoes may be connected with the relative abundance of certain biomolecules and other defining factors. Nogueira et al. (2015) reported that EVs from extremely virulent *T. cruzi* Colombiana expressed much less α-Gal epitopes than virulent strains, but it remains uncertain if EV-incorporated molecules correlates with virulence during host-parasite interaction given the condition of *in vitro* stimulation of EVs and host genetic factors, and the physiological condition within the vector. Identified virulence factors in *Leishamania* EVs include gp63, redox enzymes like tryparedoxin peroxidase, and HSPs (Montaner et al., 2014), whereas African trypanosome EVs contained and expressed serum resistance-associated (SRA) protein (Szempruch et al., 2016b). Virulence factors including the transsialidase family of glycoproteins, cruzipain, and MASPs have been found in *T. cruzi* EVs, which predicates *T. cruzi* pathogenesis (Lozano et al., 2017) and virulence (Ribeiro et al., 2018) (Table 1). Nevertheless, further clarifications on the roles of specific putative PPEV antigenic molecules and factors responsible for PPEV molecular sorting will be of tremendous addition to the study of parasitic protozoan EVs.

PPEVs could also contain specific antigenic proteins as observed in *T. gondii* exosomes which participate in parasite invasion and replication (Silva et al., 2018). Bautista-lópez et al. (2017) has also pointed out that phosphatases and membrane-bound proteins of *T. cruzi* EVs triggered Ca^2+^ signaling with lysosome mobilization and exocytosis that enhanced the formation of parasitophorous vacuoles and parasite invasion. *T. cruzi* membrane-shed vesicles contained trypomastigote surface glycoproteins (Torrecilhas et al., 2009) which may prime toll-like receptors (TLRs) containing GPI-anchors on host cells for parasite invasion (Ribeiro et al., 2018).

### PPEVs and Host Immune Responses

PPEVs can promote, re-direct, and suppress immune cell responses depending on the maturation of the immune cell, disease model, *T. brucei* EV concentration (Silverman et al., 2010b), amount of *T. cruzi* EVs (Cronemberger-Andrade et al., 2020), site of *T. cruzi* EV inoculation (Lovo-Martins et al., 2018), and time. PPEVs cannot cause infection *per se* but it can aid subsequent parasitization and diverse innate and chronic immune responses (de Souza and Barrias, 2017). *T. gondi-*derived EVs can elicit humoral and cellular immune responses separately or simultaneously in the host (Li et al., 2018b). Exacerbated immune response, in part, may occur when EVs are up-taken by immune cells and in the process elicits changes in the host cell transcriptomes leading to stronger immune cell recruitments than parasite-induced signals (Montaner et al., 2014). During infection, *T. gondi* EV-primed immune cells could acquire new membranous receptors, enzymes, and genetic material which might induce intracellular signaling (Li et al., 2018b). Thus, PPEVs are mediators of biological signals and immune responses (Fernandez-Becerra et al., 2014) (Table 2).

**Table 2:** Immune cell functional responses after interaction with PPEVs.

**Disease**	**Parasite EVs**	**Study type**	**Target cell**	**Functional response**	**References**
keratitis	Ac EVs	*in vitro*	Human	Increased IL-6 and IL-12	Lin et al., 2019
Granulomatous amoebic			Monocytes		
Meningoencephalitis					
Malaria	Pf RMVs	*in vitro*	PBMCs macrophage neutrophils	Upregulation of CD40, CD54, and CD86; decreased IL-10 IL-10 and TNF-α	Mantel et al., 2013
	Pg MPs	*in vitro*	Macrophage	Increased CD40 and TNF expression	Couper et al., 2010
	pf RMVs	*in vitro*	Macrophage/PBMCs	Activation of IL-6, IL-12, IL-1β, and IL-10	Mantel et al., 2013
	Pf EVs	*in vitro*	Monocytes	mRNA induction of CCL5, of CCL5, CXCL10, IFNα, IFNB, IFIT1	
Neosporosis	Nc EVs	*in vitro*	Macrophage	IL-12p40, TNF-α, IL-1β, IL-6	Li et al., 2018c
			BMDMs	IFN-γ, and IL-10 increased	
Toxoplamosis	Tg exosome	*in vitro*	Macrophage	IL-12, TNF-α and IFN-γ signicantly increased	Li et al., 2018b
	Tg EVs	*in vitro*	Splenocytes	Significant high IFN-γ, IL-12; CD8+ subset of T cells	Li et al., 2018c
	Tg EVs	*in vitro*	Murine	mRNA expression of IL-10	Li et al., 2018c
			Macrophage	TNF-α, iNOS up-regulated	
Trypanosomiasis	Tc EVs	*in vivo*	BMDMs	Decreased TNF-α IL-6, NO,	Lovo-Martins et al., 2018
		*in vitro*	Spleen cells	TNF-α,IL-6,IL-12p70, IFN-γ, MCP-1, IL-10^*^	
			Murine	Induction of LB and PGE_2_	
			Macrophage		
	Tc vesicles		Splenocytes, macrophages, B cell	higher TNF-α, IFN-γ, IL-6, IL-10, NO, IL-10 CD4+ and CD8+	Nogueira et al., 2015
			DC	higher T lymphocytes, TNF-α	
	Tc vesicles	*in vivo*	Splenocytes	Higher IL-10 levels, not IL-4 and NO	Torrecilhas et al., 2009
			Mice heart	induced IL-10 and IL-4 mRNA	
	Tc EVs	*in vitro*	CHO/CD14	lower IL-1β and higher IL-6 inductions, and TNF-α^*^	Cronemberger-Andrade et al., 2020
Leishamaniasis	Li exosome	*in vitro*	DC macrophage	MHC-ll basal, decresed CD40 and CD 86	Pérez-Cabezas et al., 2018
	Ld exosomes	*in vitro*	MoDCs	increased TNF-α, IL-6, IL-8 reduced CD80, CD86, HLA-DR increased IFN-γ, IL-10, IL-17	Silverman et al., 2010b
		*in vitro*	Splenocytes	higher IFN-γ, IL-4(CD4)Tcells	
			Spleen nymph node	lower IFN-γ (CD4 T cells) and Foxp3	
	Li exosomes	*in vitro*	Human	Inducted IL-10	Castelli et al., 2019
			Monocytes	Reduced IL-18	
	Lm exosomes	*in vivo*	Lymph node	Inducted IL-17a, IL-4, IL-23, INF-y	Atayde et al., 2015
	La EVs	*in vitro*	Macrophage	Increased IL-6, IL-10	Barbosa et al., 2018
		*in vitro*	B-1 cell	Increased IL-6, decreased IL-10	
Trichomaniasis	Tv exosome	*in vitro*	Macrophage	increase NO, IL-6, IL-8 IL-10, IL-17,IL-22 and TNF-α expression	Olmos-Ortiz et al., 2017
Giardiasis	Gi microvesicles	*in vitro*	Dendritic cel	CD25, T cell alloproliferation	Evans-Osses et al., 2017

*PBMCs, peripheral blood mononuclear cells; Pg, P. berghei; Ac, A. castenallii; N. caninum; Tg, T. gondii; Tc, T. cruzi; Li L, infatum; Lm, L. major; La, L. amazonensis; Ld, L. donovani; Tv, T,vaginalis; Gi, G. intestinalis; DC, dendritic cell; MoDCs, momocyte derived DC; LB, lipid body; and PGE_2_, Prostaglandin E_2_ CHO/CD14; TLR2, transfected macrophage cell line*.

* *No change in expression level*.

Components of protozoan parasite EVs that can affect innate immune response include agonists of pattern recognition receptors, mRNA, miRNAs, sRNAs, DNA, fibronectin, several pathogen-associated molecular patterns, and glycopeptidolipids (Yanez-Mo' et al., 2015; Castelli et al., 2019). The packaging of these molecules in PPEVs may prevent their recognition by the host immune system (Roditi, 2016), and the specific EV protein composition can considerably affect the phenotypic responses of cytokines (Silverman et al., 2010b). For instance, when mice were immunized with rex, a purified exosomal protein from *P. yoelii*, 83% of the mice survived the primary challenge and remained immunoprotected (Fernandez-Becerra et al., 2014). A similar down-regulation of immune cells with longer parasite survival time had been reported in mice immunized with non-lethal *P. yoelii* 17X-derived exosomes with a significant increase of reticulocytosis and changes in the parasite tropism (Martin-Jaular et al., 2011).

Twu et al. (2013) reported a potential critical role of dampening interleukin 8 (IL-8) response secreted by ectocervical cells after an exposure to *T. vaginalis* exosomes in order to establish successful chronic infections. In another instance, *T. vaginalis* exosome-like vesicles modified cytokine production in macrophages and ameliorated inflammatory process in mice model of trichomoniasis (Olmos-Ortiz et al., 2017). It has been reported also that *Leishmania* exosomes selectively induced IL-8 secretion to suppress host response (Silverman et al., 2010a). An early signal of lL-10, an anti-inflammatory cytokine, after the incubation of *T. gondi*-derived exosomes with macrophages shows that EVs promote parasite survival (Li et al., 2018a). *L*. *donovani* wild-type exosomes also promoted the secretion of IL-10 to create an infectious environment for parasite survival, but such property was not exhibited by mutant HSP100^−/−^ *L*. *donovani* exosomes (Silverman et al., 2010b), and intracellular *T. cruzi* vesicles induced local reduction of inducible nitric oxide (iNO) activity which supported higher tissue parasitism (Torrecilhas et al., 2009) (Table 2). Thus, specific PPEVs can commonly impact the phenotypic responses of cytokines during protozoan parasite infection.

During malaria infection, parasite-derived MPs, or RMVs dominantly drive macrophage activation by either causing pathological inflammation or initiating anti-malaria immune responses which contributed to the local, systemic, and EV-dose-dependent production of pro-inflammatory cytokines and chemokines (Mantel et al., 2013, 2016; Kehrer et al., 2016). iRBC-derived vesicles induced pro-inflammatory cytokines such as IL-6 and IL-1 in human bone marrow-derived endothelial cells (Mantel et al., 2016). MPs have been shown to be responsible for macrophage activation when co-cultured with iRBC MPs with the significant up-regulation of cluster of differentiation CD 40 and production of tumor necrosis factor (TNF). In the process, the induced CD40 on antigen-presenting cells primed T cells for effector functions (Couper et al., 2010). Likewise, *L. infantum* EVs recruited more macrophages and dendritic cells than did other extracellular products or the parasite (Pérez-Cabezas et al., 2018). The derived mucin-like glycoproteins and glycoinositol phospholipids in *T. cruzi* trypomastigote EV were likely responsible for the induction of inflammatory responses in macrophages (Cronemberger-Andrade et al., 2020).

*T. cruzi* vesicle-derived cruzipain has been described to enhance the production of pro-inflammatory IL-4 and IL-5 cytokines (Torrecilhas et al., 2009). Equally, *A. castellanii* EVs triggered the transcription of pro-inflammatory cytokines in monocytes (Lin et al., 2019), but no specific antigenic product was identified. Parasite specific virulent molecules in *T. cruzi* EVs induced different levels of pro-inflammatory TNF-α, IL-6, and NO responses under the same treatment (Nogueira et al., 2015). Meanwhile, the quantification of cytokine secretion by ectocervical cells demonstrated that *T. vaginalis* exosomes induced IL-6 as *T. vaginalis* and promoted acute inflammation (Twu et al., 2013). *T. gondii* exosomes have been shown to affect the progress of intracellular infections with an onward regulation of inflammatory cytokines (IL-12, IFN-γ, and TNF-α in macrophages) and Th1 responses (Li et al., 2018a). There was also a significant increase in the production of IL-4 and TNF-α by *L. amazonensis* in the presence of *L. amazonensis* EVs (Barbosa et al., 2018) (Table 2). The enhanced inflammation observed in mice co-injected with *L. major* exosomes was attributed to the possible intermediation of Th17 cells in the lymph node (Atayde et al., 2015). Remarkably, EVs from *T. cruzi* Colombiana and Y strains induced a more pro-inflammatory reaction than those of YuYu and CL-14 strains (Nogueira et al., 2015).

Major humoral immune response elicited by PPEVs has been reported after mice immunization with *T. gondii* exosomes in which a high level of total IgG reminiscent of Th1 cells was detected (Li et al., 2018a). As well, exosomes obtained from *P. yoelii*-infected mouse reticulocytes elicited IgG2a and IgG2b isotype antibodies that recognized *Plasmodium*-infected RBCs (Martin-Jaular et al., 2011). MASPs in *T. cruzi* trypomastigote EVs triggered a rapid humoral IgM response but limited IgG class-switching during infection (Bautista-lópez et al., 2017). A rather significant role of EVs in immune modulation was seen with *T. cruzi*-derived EVs, which induced lipid body formation and prostaglandin E_2_ in murine macrophages (Lovo-Martins et al., 2018) (Table 2).

Interaction of PPEVs and protozoan parasites can heighten immune responses and pathogenesis. In respect to this, Lovo-Martins et al. (2018) had shown that the pre-inoculation of *T. cruzi* trypomastigote vesicles before parasite infection produced IL-4 which was dependent on parasite strain. Also, the inoculation of *T. cruzi*-derived EVs following *T. cruzi* infection resulted in the induction of high levels of TNF-α, interferon gamma (IFN-γ), monocyte chemoattractant protein-1 (MCP-1), and IL-6 cytokines (Lovo-Martins et al., 2018). *T. cruzi* trypomastigote-derived EVs elicited increase in TNF-α and IL-6 release in bone-marrow macrophage response (Choudhuri and Garg, 2020). This establishes the concept that a protozoan parasite and its derived EVs may work in tandem to establish infection. Also, EV pro-parasitic actions are progressively being shown to be strain-specific. Ribeiro et al. (2018) reported that macrophages pre-treated with EVs from *T. cruzi* Y strain showed increased trypomastigote invasion, whereas pre-treatment with EVs from the *T. cruzi* YuYu strain displayed increased intracellular parasite proliferation. However, inoculation of mice with EVs of *T. cruzi* YuYu and CL-14 strains without subsequent infection did not stimulate inducible nitric oxide in the macrophage or spleenocytes, and EVs of the *T. cruzi* Y strain induced a local reduction of iNOs with subsequently higher tissue parasitism (Nogueira et al., 2015).

Secreted molecules in PPEVs may also be deployed by a parasite for immune evasion or to avoid extracellular degradation (Caeiro et al., 2018). On this basis, EVs from an intracellular and extracellular protozoan parasites promote growth and induce host immune evasion by manipulating the microenvironment for adaptive responses or inhibition of inflammation (Mantel and Marti, 2014). Kinases and glycosidases found in EVs of *A. castellanii* could act on extracellular matrix to favour the escape of *A. castellanii* from immune cells (Gonçalves et al., 2018). It has been speculated that *T. brucei* microvesicles may serve as antigenic epitopes deployed by the parasite to overwhelm the host immune system (Geiger et al., 2010), but more importantly, *L. infantum* promastigotes interacted with their extracellular products to initiate eventual immune evasion by modulating bone marrow-derived DC (BMDC) and impairing macrophage ability to eliminate *L. infantum* (Pérez-Cabezas et al., 2018). GPI-anchored tetraspanin proteins of EVs may also protect pathogenic protozoa from complement-mediated lysis as they support parasite evasion (Lozano et al., 2017) (Table 2).

The release of immune molecules during protozoan parasite infections has a correlation with different immune pathways. *T. cruzi* EVs stimulated the JAK/STAT signaling pathway through cytokine receptor-linkage wherein there were expressions of STAT1 and STAT3 mRNAs in macrophages (Cronemberger-Andrade et al., 2020). Available data suggest that *N. caninum* EV could activate the mitogen-activated protein kinases (MAPK) signaling pathway in bone marrow-derived macrophages (BMDMs) through a component of secretory proteins in its EVs by phosphorylation of mitogen-activated proteins (P38, ERK, and JNK) via TLR 2. Also, EVs of *T. cruzi* Y strain and Colombian strain activated MAPKs via TLR2 in peritoneal macrophages (Nogueira et al., 2015). Prior exposure of *T. cruzi* Y strain trypomastigote EVs to human macrophages transfected with TLR2 expressed CD25 and activated NF-κB via TLR2 (Cronemberger-Andrade et al., 2020). Meanwhile, TLR2 might be activated by *N. caninum* EVs in BMDMs because it contains some pathogen-associated molecular patterns (PAMPs) (Li et al., 2018c). A similar work showed that *T. gondii* exosomes induced elevated expression of JNK mRNA, activated the nuclear translocation of phosphorylated JKN-protein, and eventually activated the MAPK pathway (Li et al., 2018b). An entirely novel TLR-4/MyD88-mediated activation of macrophages by microparticles of *Plasmodium-*parasitized RBCs has been reported in malaria inflammatory responses (Couper et al., 2010). In particular, *T. cruzi*-derived EVs from different strains have been shown to activate ERK 1/2, JNK, and p38 via its protein and α-galactosyl contents (Nogueira et al., 2015). RNA and gDNA contained in *P. falciparum-*iRBCs EVs translocate into the monocytes to stimulate STING-TBK1 (protein kinase)-IRF3 (transcription factor 3)-dependent gene induction (Sisquella et al., 2017). The MAPK pathway is essential for the production of inflammatory cytokines in parasitic infections, but the translational roles of PPEVs in MAPK, STING, and TLR stimulations need to be further probed.

## Perspectives and Conclusion

Serum-starved culture has often been used to induce PPEVs, but the exact process of EV secretion in appropriate hosts might not have been comprehensively captured given anatomical, genetical, and physiological interplays in hosts and vectors. Can the inability of protozoan parasites to secrete EVs in certain hosts/vectors justify the existence of paratenic hosts or the mark of parasite dead end? PPEVs cause cellular distress and orchestrate multiple pathophysiological processes. Are there functional selective secretion mechanisms for PPEVs or causal mechanisms of genetic/epigenetic reprogramming by which PPEVs confer virulence on non-pathogenic species? Additionally, the biological process that grounds the signaling events of PPEVs in parasite-parasite interaction and epigenetic effects of EV expulsion on protozoan parasites needs to be investigated.

Wittingly, heterogeneous population of PPEVs requires functional and reproducible sorting into distinct sub-populations. Asymmetric flow field-flow fractionation has been used to separate distinct vesicles called exomere from EVs aggregates (Zhang et al., 2018) and it stands as a promising technology to separate PPEVs into distinct sub-types. In addition, lipids play important roles in EV biosynthesis, but studies aimed at elucidating PPEV lipidomics are underrepresented, and the specific roles of sugar molecules during internalization or adhesion of PPEVs need validation because sugar can specifically bind to lectin-like receptors on parasites. Also, EV-associated polysaccharides and lipid moiety are important therapeutic targets as they can induce protective and pro-inflammatory immune responses (Nogueira et al., 2015; Kuipers et al., 2018).

Inflammasomes are molecular structures of the innate immune system which induce inflammation in response to infectious microbes and molecules (Abal, 2017; Cypryk et al., 2018), but their roles have not been established in inflammatory responses to PPEVs. Studies on antigenic regions of PPEV proteins and specific immune response (Pablos et al., 2016) require further consideration especially by *in vivo* methods because parasite molecules are much more expressed in definitive hosts (Ramírez-Flores et al., 2019) (Table 2). Considering the physiological stress under which PPEVs and tsRNAs are formed and the biological functions of tsRNA in post-transcriptional regulations (Dou et al., 2019), the exact roles and vesicular loading process of tsRNAs, non-coding RNAs, and DNA into PPEVs call for elucidation.

From this review, it is obvious that the composition and function of PPEVs are a reflection of the species of origin and the activating stimuli. PPEVs can be internalized by host cells and protozoan parasites using contiguous receptor-dependent and receptor-independent mechanisms to cause various cellular distresses and to provide genetical cues. PPEVs have been shown to induce differential cytokine expression depending on cell type, infection model, dose and origin of EVs. In extreme cases, PPEVs may present a similar effect as protozoan parasites or, at least, act in concert. The secretion of EVs by protozoan parasites comparatively represents parasite constitutive encryptions with which they harness developmental stimuli, nutritional materials, digestive enzymes, and control of maturation. We have only unveiled the phenomenal responses of pathogenic protozoa to stressors, secretion and internalization of EVs, and vesicular peculiarities with the hope that it would help to address fundamental questions on parasite biology.

## Author Contributions

JC proposed the contents and paper frame and provided critical feedback. JO drafted the manuscript. All authors contributed to the article and approved the submitted version.

## Conflict of Interest

The authors declare that the research was conducted in the absence of any commercial or financial relationships that could be construed as a potential conflict of interest.
